# Supplementary material for: The yeast ISW1b ATP-dependent chromatin remodeler is critical for nucleosome spacing and dinucleosome resolution
Source: Sci Rep. 2021 Feb 18;11:4195. doi: 10.1038/s41598-021-82842-9 (PMC7892562; doi:10.1038/s41598-021-82842-9)
Supplement: Supplementary file 1 — Supplementary Information 1. [file 41598_2021_82842_MOESM1_ESM.pdf]

9 February 2021

The yeast ISW1b ATP-dependent chromatin remodeler is critical for nucleosome spacing and dinucleosome resolution

Peter R. Eriksson and David J. Clark

Supplementary Material

Supplementary Figures S1 – S5

Supplementary Tables S1 and S2

Supplementary References

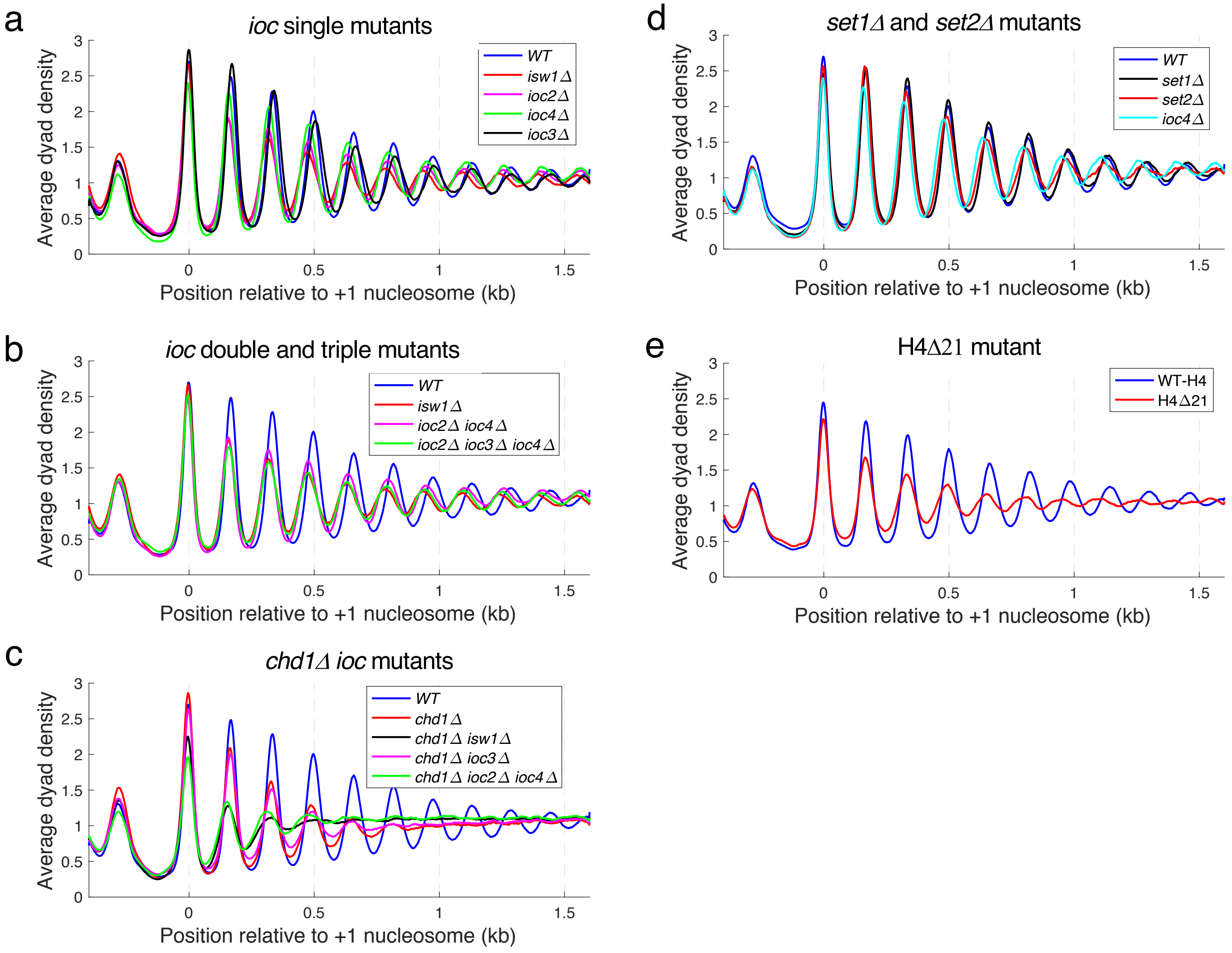

f

	Nucleosome spacing on all genes					*Nucleosome spacing on genes > 2 kb*					*Change in average spacing*
	**Replicate A**	**Replicate B**	**Average (bp)**	**WT - mutant (bp)**		**Replicate A**	**Replicate B**	**Average (bp)**	**WT - mutant (bp)**		**Long genes - All genes (bp)**
Wild type	164.6	164.6	**164.6**	-	Wild type	165.0	165.1	**165.1**	-	Wild type	**0.5**
isw1	159.2	159.7	**159.5**	**5.2**	*isw1*	158.3	158.6	**158.5**	**6.7**	*isw1*	**-1.0**
ioc2	162.3	161.4	**161.9**	**2.7**	*ioc2*	158.6	158.6	**158.6**	**6.5**	*ioc2*	**-3.3**
ioc4	162.6	161.9	**162.3**	**2.3**	*ioc4*	160.0	159.7	**159.9**	**5.3**	*ioc4*	**-2.4**
ioc2 ioc4	162.7	161.7	**162.2**	**2.4**	*ioc2 ioc4*	159.4	159.0	**159.2**	**5.9**	*ioc2 ioc4*	**-3.0**
ioc2 ioc3 ioc4	160.0	159.6	**159.8**	**4.8**	*ioc2 ioc3 ioc4*	158.9	157.9	**158.4**	**6.7**	*ioc2 ioc3 ioc4*	**-1.4**
ioc3	166.0	166.5	**166.3**	**-1.7**	*ioc3*	166.6	167.2	**166.9**	**-1.8**	*ioc3*	**0.6**
chd1	163.8	163.2	**163.5**	**1.1**	*chd1*	162.2	162.1	**162.2**	**3.0**	*chd1*	**-1.4**
chd1 ioc3	163.4	163.3	**163.4**	**1.2**	*chd1 ioc3*	164.3	163.7	**164.0**	**1.1**	*chd1 ioc3*	**0.6**
chd1 ioc2 ioc4	ND	ND	**ND**	**ND**	*chd1 ioc2 ioc4*	ND	ND	**ND**	**ND**	*chd1 ioc2 ioc4*	**ND**
chd1 isw1	ND	ND	**ND**	**ND**	*chd1 isw1*	ND	ND	**ND**	**ND**	*chd1 isw1*	**ND**
set1	165.1	165.1	**165.1**	**-0.5**	*set1*	164.6	164.4	**164.5**	**0.6**	*set1*	**-0.6**
set2	164.9	163.1	**164.0**	**0.6**	*set2*	163.5	162.9	**163.2**	**1.9**	*set2*	**-0.8**
Wild type H4	165.8	165.9	**165.9**	-	Wild type H4	165.8	165.9	**165.9**	-	Wild type H4	**0.0**
H4del21	162.9	162.1	**162.5**	**3.4**	H4del21	163.5	163.1	**163.3**	**2.6**	H4del21	**0.8**

Supplementary Fig. S1. Nucleosome phasing on genes longer than 2 kb. Average nucleosome phasing in the various mutants on the 1616 genes longer than 2 kb from transcription start site (TSS) to transcript termination site (TTS). On long genes, promoter-proximal nucleosome organisation is less likely to be influenced by downstream TTSs or downstream TSSs. (**a-e**) Data for Replicate A. (**f**) Summary of nucleosome spacing for the +1 to +5 nucleosomes on long genes compared with all genes. ND = not determined because the chromatin is too disrupted.

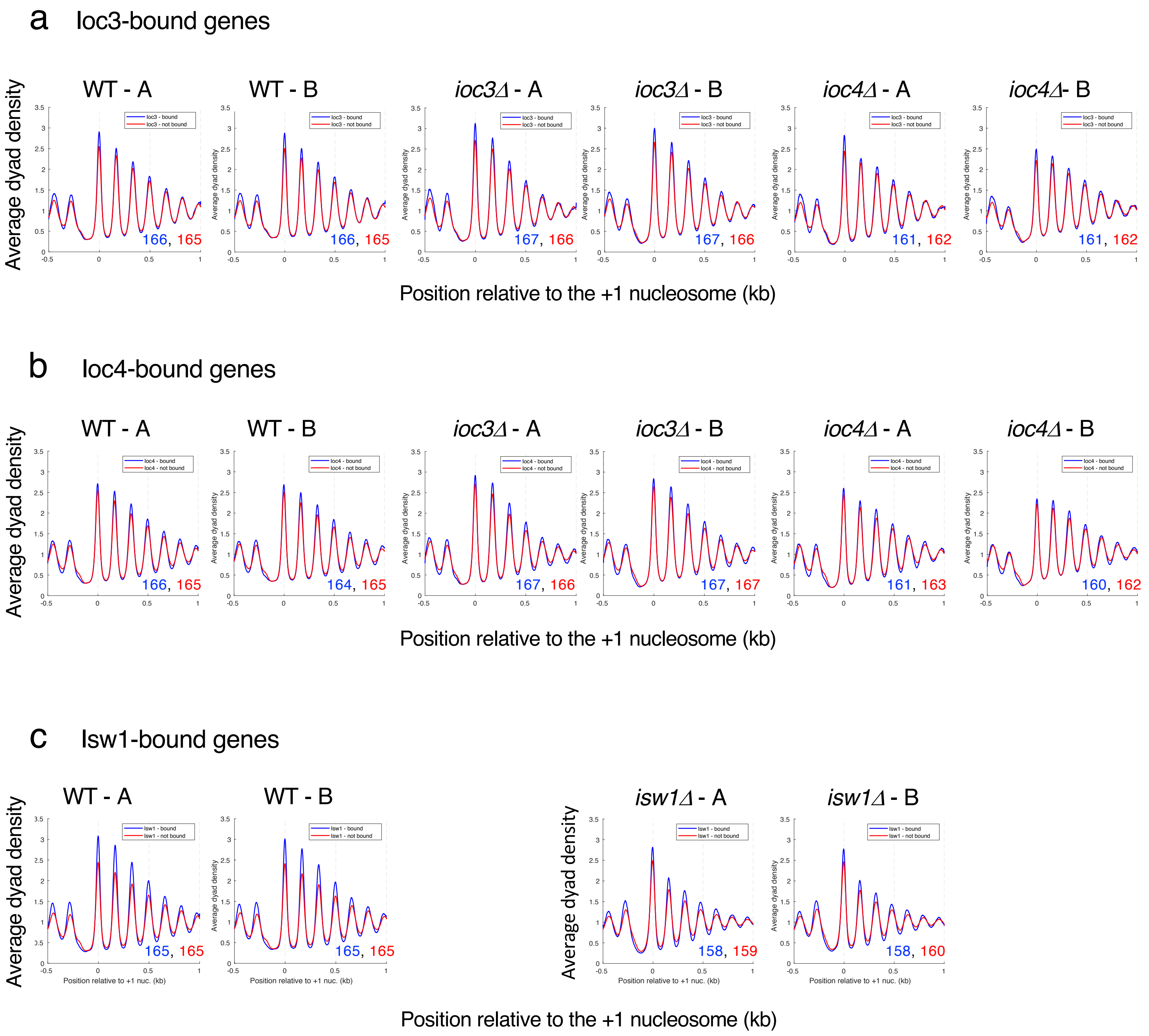

Supplementary Fig. S2. The chromatin organisations of Ioc3-bound, Ioc4-bound and Isw1-bound genes are not significantly different from the unbound genes. Genes enriched for ISW1a (Ioc3-bound), ISW1b (Ioc4-bound) and both complexes (Isw1-bound) are defined by ChIP-seq data [1]. Average nucleosome dyad density plots for bound and unbound genes: (**a**) Ioc3-bound (blue line) and non-bound (red line) genes in wild type (WT) and *ioc3Δ* cells. (**b**) Ioc4-bound (blue line) and non-bound (red line) genes in WT and *ioc4Δ* cells. **(c**) Isw1-bound (blue line) and non-bound (red line) genes in WT and *isw1Δ* cells. All 5770 yeast genes were aligned on the midpoints of their +1 nucleosomes. The dyad distribution was normalised to the global average (set at 1). Two biological replicate experiments (A and B) are shown. The average spacing in bp is shown for bound genes (blue text) and non-bound genes (red text) in the bottom right corner (measured by regression analysis of the first 5 nucleosome peaks, beginning with the +1 nucleosome).

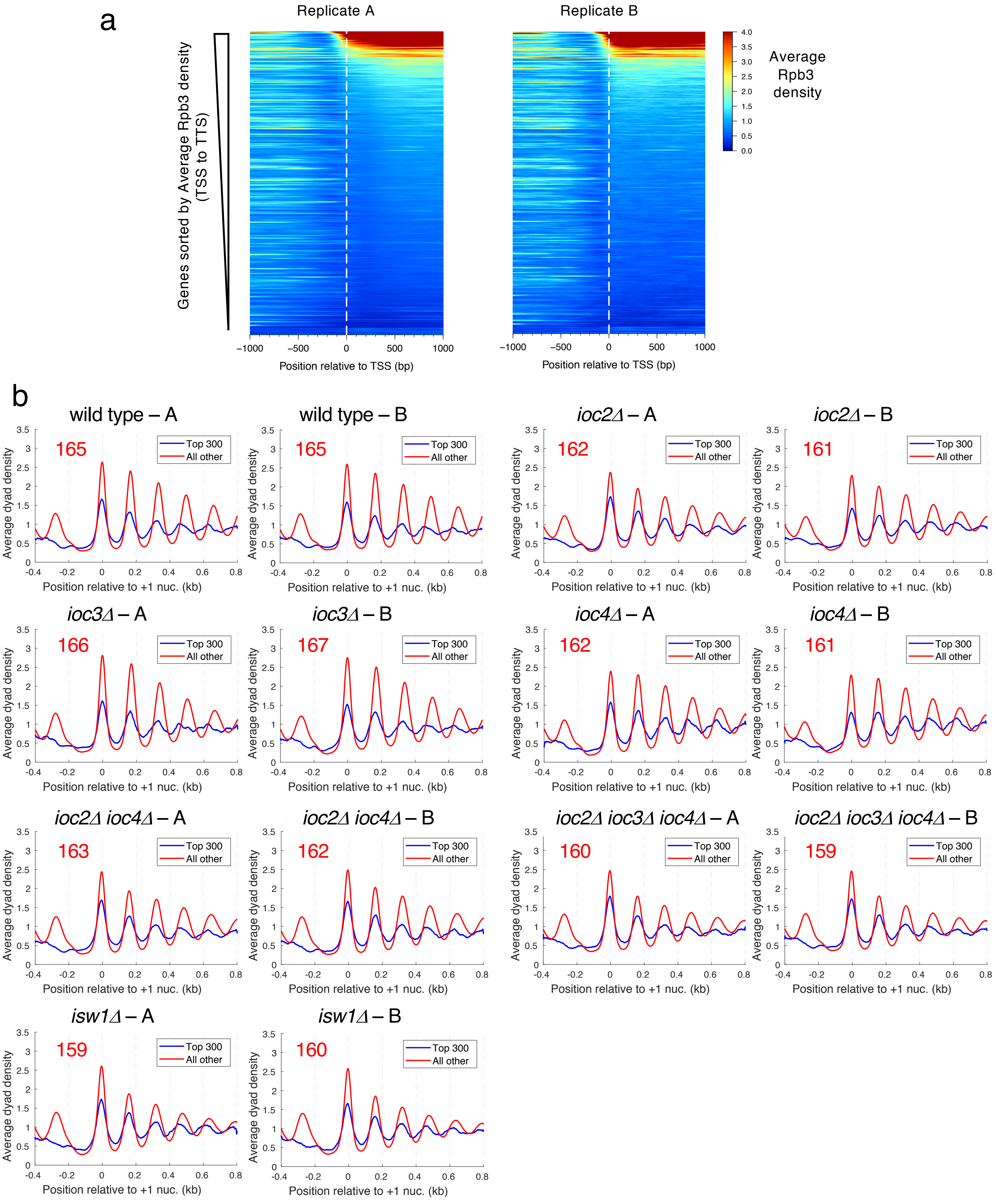

Supplementary Fig. S3, *continued on the next page*.

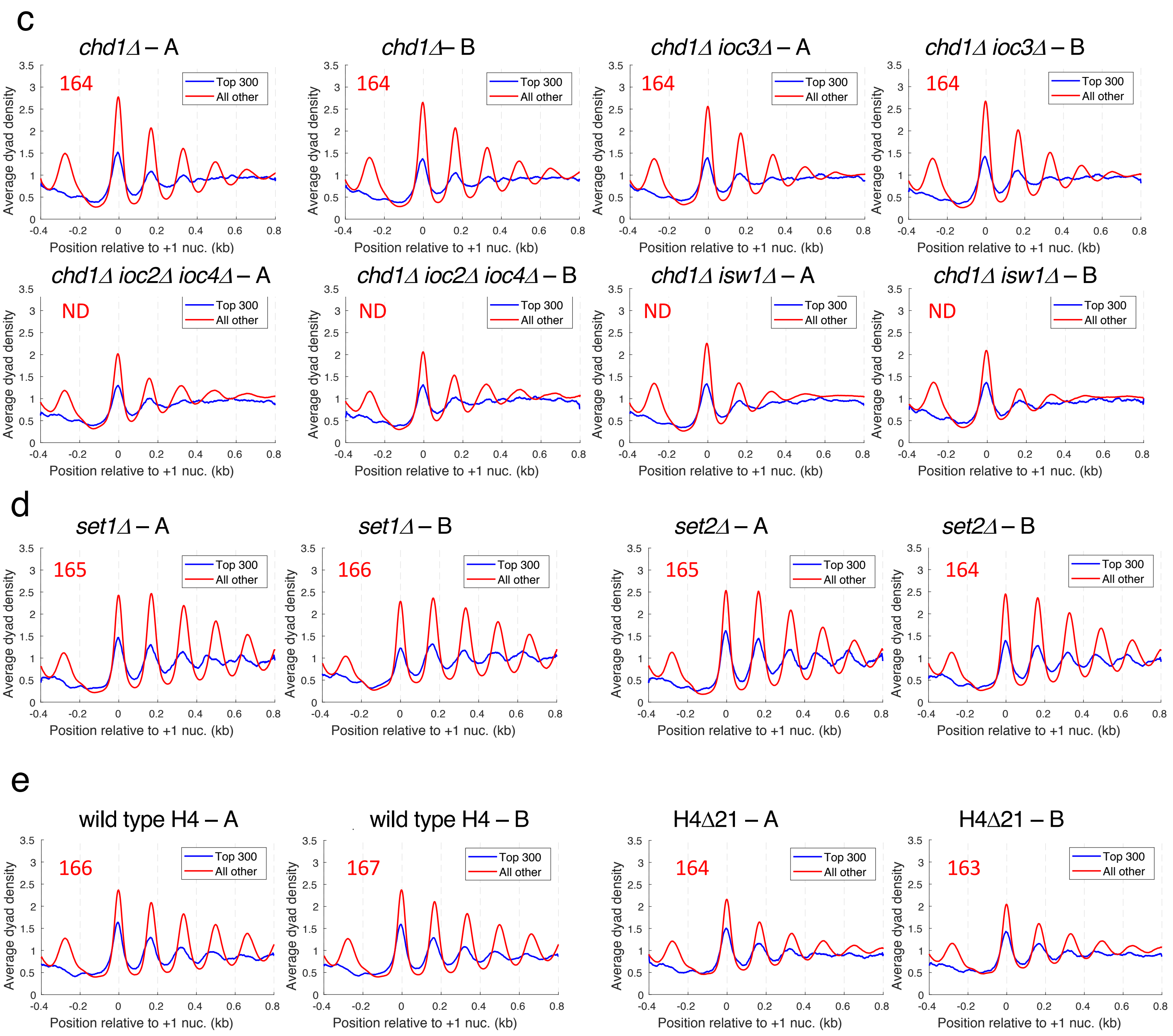

Supplementary Fig. S3. Chromatin organisation of the most highly transcribed genes compared to the remaining genes. (**a**) Heat map analysis of genic Pol II density. ChIP-seq data for the Rpb3 subunit of Pol II in wild type cells (biological replicate data from [2]). The average Pol II density for each of the 5770 genes was computed from transcription start site (TSS) to transcript termination site (TTS). Genes were aligned on the TSS and sorted from high to low Rpb3 density. (**b-e**) Average nucleosome dyad density plots for the 304 most active genes (defined by Rpb3 density > 4 times the genomic average: blue line) compared with the remaining 5470 genes (red line). Genes were aligned on the midpoints of their +1 nucleosomes. The dyad distribution was normalised to the global average (set at 1). Two biological replicate experiments (A and B) are shown in separate panels. The average spacing in bp is shown for the top 300 active genes (blue text) and the other genes (red text), as measured by regression analysis of the first 5 nucleosome peaks, beginning with the +1 nucleosome. (**b**) Wild type (WT), *ioc2Δ, ioc3Δ ioc4Δ, ioc2Δ, ioc2Δ ioc4Δ, ioc2Δ ioc3Δ ioc4Δ* and *isw1Δ*. (**c**) *chd1Δ, chd1Δ ioc3Δ, chd1Δ ioc2Δ ioc4Δ* and *chd1Δ isw1Δ*. (**d**) *set1Δ* and *set2Δ*. (**e**) Wild type H4 and H4Δ21.

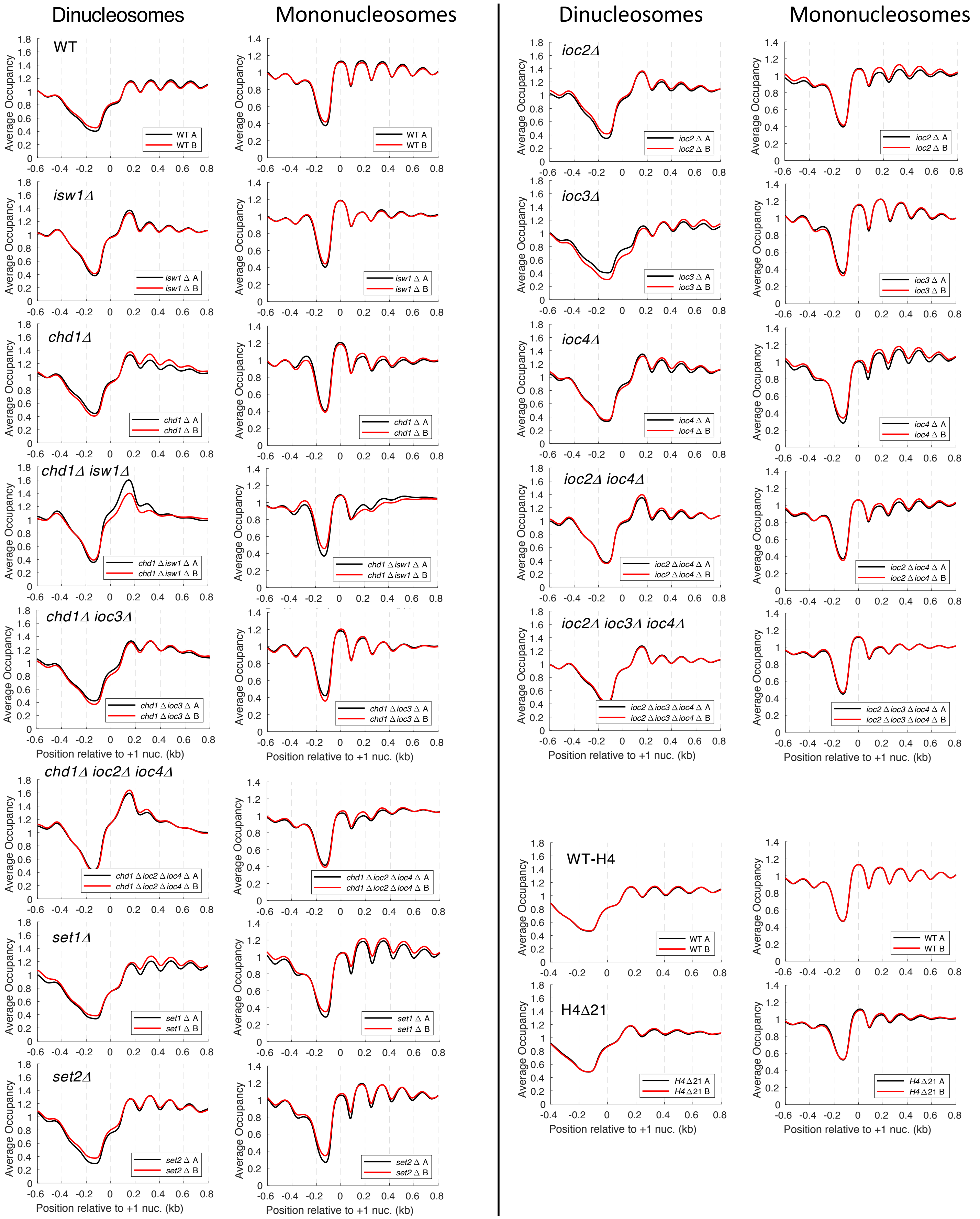

Supplementary Fig. S4. Average mononucleosome and dinucleosome occupancy (coverage) plots for all genes. Comparison of biological replicate experiments. All 5770 yeast genes were aligned on the midpoint of their average +1 nucleosome position. Occupancy was normalised to the global average (set at 1) for dinucleosomes (250-350 bp; left panels) or mononucleosomes (120-180 bp; right panels). Note different y-axis scales are used in the dinucleosome and mononucleosome plots. Replicate A (black line); replicate B (red line).

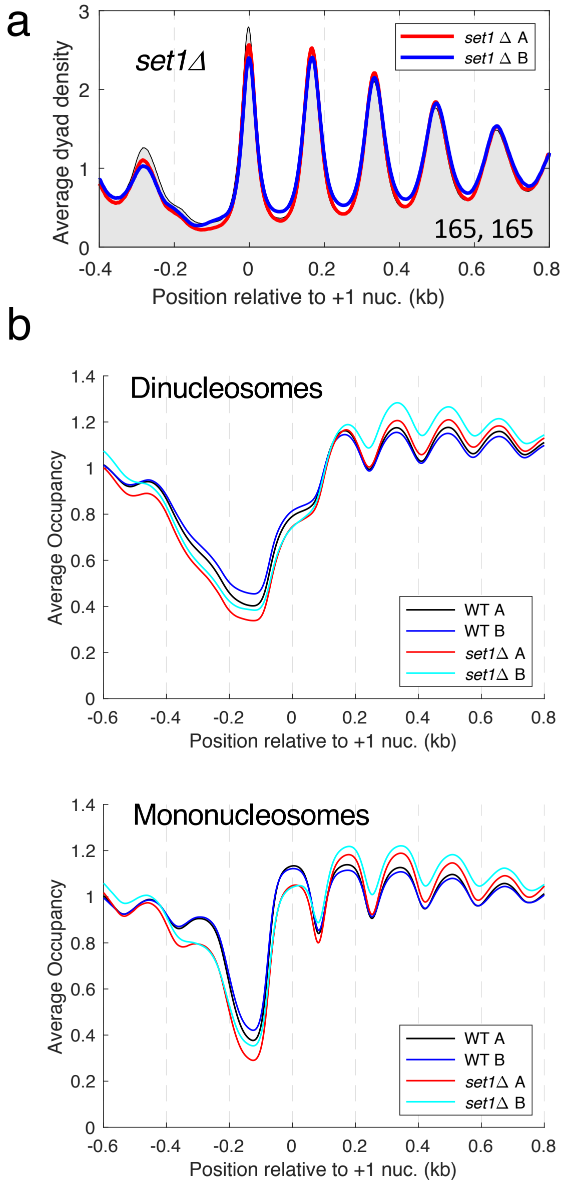

Supplementary Fig. S5. Set1 has little effect on global chromatin organisation. (**a**) Average nucleosome dyad density plot for all genes in *set1Δ* cells. Wild type replicate A is shown as a black line with grey fill. Two biological replicate experiments are shown: A (red line); B (blue line). The average spacings (bp) for replicates A and B are shown (bottom right). (**b**) Occupancy plots for dinucleosomes and mononucleosomes in *set1Δ* and wild type (WT) cells (see legend to Fig. 3).

Supplementary Table S1. Primers used in this study.

1770 (H4Δ21)	ACTGTTCCGAGCGCTTCT
1771 (H4Δ21)	[5’-phosphate]-CATATTTTACTATATTATATTTGTTGCTTG
1773 (H4Δ21)	CGAATTGGAGCTCGGTAC
1812 (H4Δ21)	[5’-phosphate]-CTAAGAGATAACATCCAAGGTATTAC
1914 (set2)	AAAACTGCATAGTCGTGCTGTCAAACCTTTCTCCTTTCCTGGTTGTTGTTTTACGTGATCCGGATCCCCGGGTTAATTAA
1915 (set2)	ACAAGACTTCCTTTGGGACAGAAAACGTGAAACAAGCCCCAAATATGCATGTCTGGTTAAGAATTCGAGCTCGTTTAAAC
1916 (set2)	TCGCAGTAATAAGGACAGTATCCG
1917 (set2)	GCAGTTAACATGTAAATTTCCCGAT
1918 (ioc3)	TTCACAAGATAACTACGACCCTCC
1919 (ioc3)	GATAACAGTATGCCGTTTTACCAC
1922 (ioc4)	TTCTGGTTTGTTTCTTTGCCTCA
1923 (ioc4)	CATATACGGTGTTAAGATGATGACA
1926 (set1)	AGGTTCATTAAATGCTTGGCTT
1927 (set1)	TTCTGGAGCGTATCTAATTGCTTG
1961 (ioc2)	GTGAATCAGCGATCGATGAGAACGAAGAGAACTAGGAGTACAAGAAATGTAGGAGCGAGCTTGTTCTTGATTTGTGCCCCGTA
1962 (ioc2)	ATGAAGAACCAGTTGATATATATTTTACTATATACTTTTGCTGTGTATTTCTATATGAGGTGGTTTCAGGGTCCATAAAGC
1965 (ioc2)	AGTGAAATACTTAACAATTGAGCTG
1966 (ioc2)	GCTCCTACATTTCTTGTACTCCT

Supplementary Table S2. Yeast strains used in this study. All are W303 *RAD5* background [3].

Strain	*Genotype*	*Reference*
YDC111	*MATa ade2-1 can1-100 leu2-3,112 trp1-1 ura3-1*	4
YTT186	*MATa ade2-1 can1-100 his3-11,15 leu2-3,112 trp1-1 ura3-1 isw1Δ::ADE2*	5
YTT196	*MATα ade2-1 can1-100 his3-11,15 leu2-3,112 trp1-1 ura3-1 isw2Δ::LEU2*	5
YTT645	*MATa ade2-1 can1-100 his3-11,15 leu2-3,112 trp1-1 ura3-1 ioc3Δ::KanMX*	Tsukiyama Lab (unpub.)
YTT827	*MATa ade2-1 can1-100 his3-11,15 leu2-3,112 trp1-1 ura3-1 ioc4Δ::HPH1*	Tsukiyama Lab (unpub.)
YTT1986	*MATα ade2-1 can1-100 his3-11,15 leu2-3,112 trp1-1 ura3-1 set1Δ::NAT1*	Tsukiyama Lab (unpub.)
YJO482	*MATa ade2-1 can1-100 his3-11,15 leu2-3,112 trp1-1 ura3-1 chd1Δ::HIS3MX6*	2
YJO484	*MATa ade2-1 can1-100 his3-11,15 leu2-3,112 trp1-1 ura3-1 isw1Δ::ADE2 chd1Δ::HIS3MX6*	2
YJO486	*MATa ade2-1 can1-100 his3-11,15 leu2-3,112 trp1-1 ura3-1 isw2Δ::LEU2 chd1Δ::HIS3MX6*	2
YJO487	*MATα ade2-1 can1-100 his3-11,15 leu2-3,112 trp1-1 ura3-1 isw2Δ::LEU2 chd1Δ::HIS3MX6*	This study
YJO505	*MATa/α ade2-1/ade2-1 can1-100/can1-100 his3-11,15/his3-11,15 leu2-3,112/leu2-3,112 trp1-1/trp1-1 ura3-1/ura3-1 ISW1/isw1Δ::ADE2 ISW2/isw2Δ::LEU2 RSC8/GALp-RSC8::KanMX CHD1/chd1Δ::HIS3MX6*	2
YPE600	*MATa/α ade2-1/ade2-1 can1-100/can1-100 his3-11,15/HIS3 leu2-3,112/leu2-3,112 trp1-1/ trp1-1 ura3-1/ura3-1 isw2Δ::LEU2*	This study
YPE606	*MATa ade2-1 can1-100 leu2-3,112 trp1-1 ura3-1 ioc3Δ::KanMX*	This study
YPE607	*MATα ade2-1 can1-100 leu2-3,112 trp1-1 ura3-1 ioc3Δ::KanMX*	This study
YPE608	*MATa ade2-1 can1-100 leu2-3,112 trp1-1 ura3-1 ioc4Δ::HPH1*	This study
YPE636	*MATa ade2-1 can1-100 leu2-3,112 trp1-1 ura3-1 ioc2Δ::URA3*	This study
YPE654	*MATa ade2-1 can1-100 leu2-3,112 trp1-1 ura3-1 ioc2Δ::URA3 ioc3Δ::KanMX*	This study
YPE655	*MATa ade2-1 can1-100 leu2-3,112 trp1-1 ura3-1 ioc2Δ::URA3 ioc4Δ::HPH1*	This study
YPE657	*MATa ade2-1 can1-100 leu2-3,112 trp1-1 ura3-1 ioc2Δ::URA3 ioc3Δ::KanMX ioc4Δ::HPH1*	This study
YPE712	*MATa ade2-1 can1-100 his3-11,15 leu2-3,112 trp1-1 ura3-1 chd1Δ::HIS3MX6 ioc3Δ::KanMX6*	This study
YPE715	*MATa ade2-1 can1-100 his3-11,15 leu2-3,112 trp1-1 ura3-1 chd1Δ::HIS3MX6 ioc2Δ::URA3 ioc4Δ::HPH1*	This study
YPE602	*MATa ade2-1 can1-100 leu2-3,112 trp1-1 ura3-1 set1Δ::NAT1*	This study
YPE604	*MATa ade2-1 can1-100 leu2-3,112 trp1-1 ura3-1 set2Δ::TRP1*	This study
ROY1281	*MATα lys2 trp1 his3 leu2 ura3 hhf1-hht1Δ::LEU2 hhf2-hht2Δ::HIS3 pCC67*	6
YDC507	*MATα lys2 trp1 his3 leu2 ura3 hhf1-hht1Δ::LEU2 hhf2-hht2Δ::HIS3 p730*	This study
YDC101	*MATα lys2 trp1 his3 leu2 ura3 hhf1-hht1Δ::LEU2 hhf2-hht2Δ::HIS3 p368*	This study

Supplementary References

1. Yen, K., Vinayachandran, V., Batta, K., Koerber, R. T. & Pugh, B. F. Genome-wide nucleosome specificity and directionality of chromatin remodelers. *Cell* **149,** 1461–1473 (2012).

2. Ocampo, J., Chereji, R. V., Eriksson, P. R. & Clark, D. J. The ISW1 and CHD1 ATP-dependent chromatin remodelers compete to set nucleosome spacing *in vivo*. *Nucleic Acids Res*. **44,** 4625–4635 (2016).

3. Zhao, X., Muller, E. G. & Rothstein, R. A suppressor of two essential checkpoint genes identifies a novel protein that negatively affects dNTP pools. *Mol. Cell* **2,** 329–340 (1998).

4. Kim, Y., McLaughlin, N., Lindstrom, K., Tsukiyama, T. & Clark, D. J. Activation of *Saccharomyces cerevisiae HIS3* results in Gcn4p-dependent, SWI/SNF-dependent mobilisation of nucleosomes over the entire gene. *Mol. Cell. Biol*. **26,** 8607–8622 (2006).

5. Tsukiyama, T., Palmer, J., Landel, C. C., Shiloach, J. & Wu, C. Characterization of the imitation switch subfamily of ATP-dependent chromatin-remodeling factors in *Saccharomyces cerevisiae*. *Genes Dev*. **13,** 686–697 (1999).

6. Dhillon, N. & Kamakaka, R. T. A histone variant, Htz1p, and a Sir1p-like protein, Esc2p, mediate silencing at HMR. *Mol. Cell* **6,** 769–780 (2000).
