# Supplementary material for: The yeast ISW1b ATP-dependent chromatin remodeler is critical for nucleosome spacing and dinucleosome resolution
Source: Sci Rep. 2021 Feb 18;11:4195. doi: 10.1038/s41598-021-82842-9 (PMC7892562; doi:10.1038/s41598-021-82842-9)
Supplement: Supplementary file 2 — Supplementary Information 2. [file 41598_2021_82842_MOESM2_ESM.zip › Eriksson_Clark_Supplemental_Code/Eriksson_Clark_Rscript_README.pdf]

20 August 2020

'R' script for analysis of MNase-seq data:

Written by Razvan Chereji (Ocampo et al. (2019) Genome Research 29, 407).
Adapted here by David Clark to create heatmaps with genes sorted by Pol II (Rpb3) density.

The script was run on the NIH Biowulf supercomputer as a series of swarm jobs.
1. Aligns the sequencing data using Bowtie2 and creates bam files from paired end data (R1 and R2.fastq files).
2. Creates length histograms (plots and .csv files).
3. Creates bigwig files for browser viewing.
4. Creates phasing profiles relative to the average +1 nucleosome dyad in wild type cells: Dyads (midpoints) and occupancy (coverage) plots and .csv files containing the data.
5. Creates occupancy and dyad heatmaps sorted by Rpb3 level.
6. Creates regression plots using 5 nucleosome peaks with the corresponding dyad profile.
Output examples for wild type replicate A are included here.

Notes
1. The fastq filenames may need to be adjusted (they must be filename.R1.fastq/filename.R2.fastq).
2. To change the number of nucleosomes for regression analysis, set "nucRank" and "peakLoc" = "1:n" for 'n' nucleosomes (process_MNase_seq_bed_file_Plus1.R, lines 373 and 375).

In your directory:
default_parameters_MNase_seq.cfg
[A text file to specify Bowtie2 alignment parameters and the DNA length range: mononucleosomes = 120-180 bp or dinucleosomes = 250-350 bp. Adjust as required.]

Step1_Start_MNase_seq_workflow_Plus1.sh

process_MNase_seq_bed_file_Plus1.R

sacCer3_annotations_JO_Dave.csv
[Contains +1 nucleosome positions and Rpb3 ChIP-seq data in wild type cells (Ocampo et al. (2016) *Nucl. Acids Res.* 44, 4625) for every yeast gene.]

Folder named: fastq_files [contains the *unzipped* .fastq files]

To run, type: ./Step1_Start_MNase_seq_workflow_Plus1.sh
