# Supplementary figures and images for: The yeast ISW1b ATP-dependent chromatin remodeler is critical for nucleosome spacing and dinucleosome resolution
Source: Sci Rep. 2021 Feb 18;11:4195. doi: 10.1038/s41598-021-82842-9 (PMC7892562; doi:10.1038/s41598-021-82842-9)

# 111P\_1\_WT\_50U

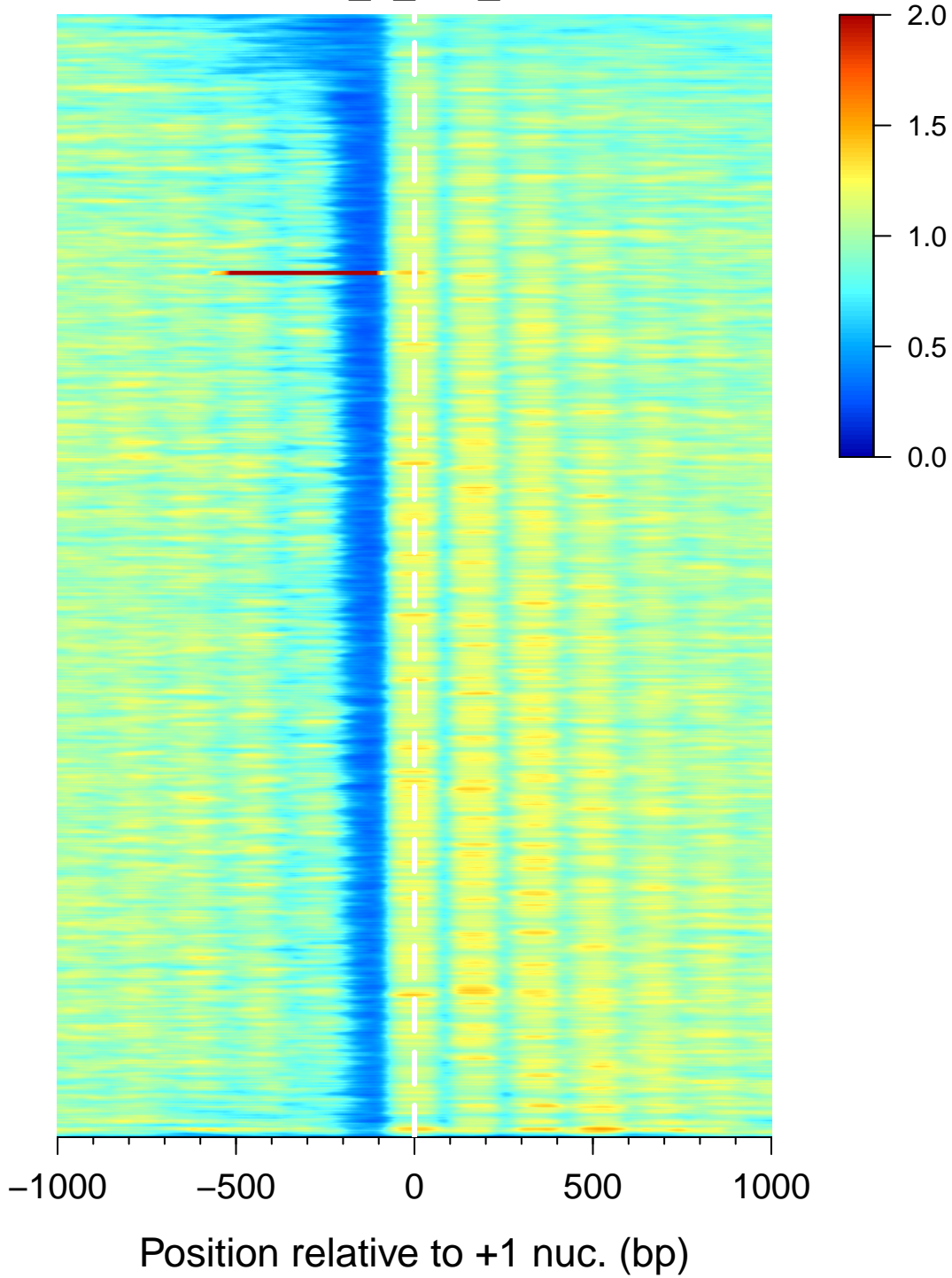

Supplement: Supplementary file 2 — Supplementary Information 2. [file 41598_2021_82842_MOESM2_ESM.zip › Eriksson_Clark_Supplemental_Code/Output_Examples/Heatmap_Occ_Plus1.111P_1_WT_50U.120_180.Rpb3_sort.pdf]

# 111P\_1\_WT\_50U

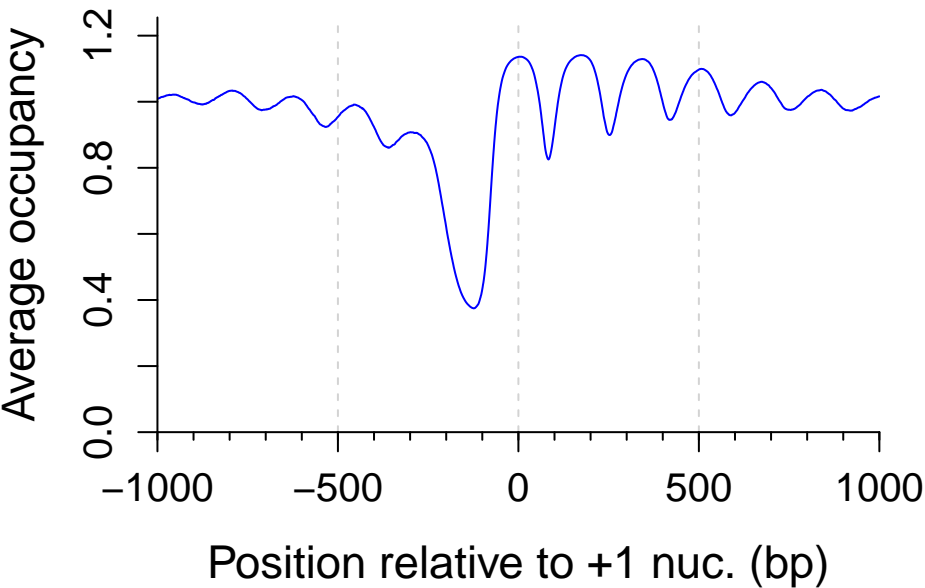

Supplement: Supplementary file 2 — Supplementary Information 2. [file 41598_2021_82842_MOESM2_ESM.zip › Eriksson_Clark_Supplemental_Code/Output_Examples/Avg_Occ_Plus1.111P_1_WT_50U.120_180.pdf]

# 111P\_1\_WT\_50U

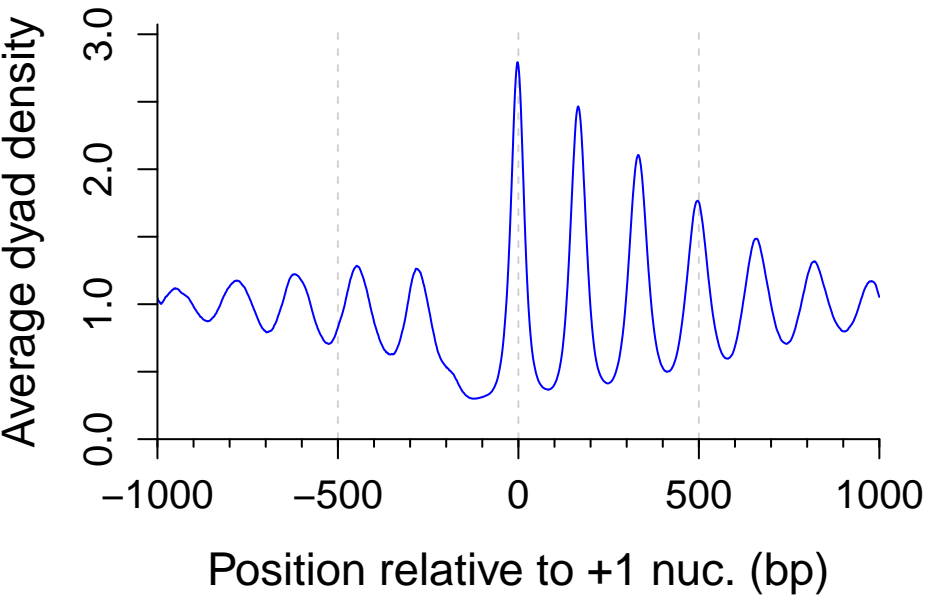

Supplement: Supplementary file 2 — Supplementary Information 2. [file 41598_2021_82842_MOESM2_ESM.zip › Eriksson_Clark_Supplemental_Code/Output_Examples/Avg_Dyads_Plus1.111P_1_WT_50U.120_180.pdf]

# 111P\_1\_WT\_50U

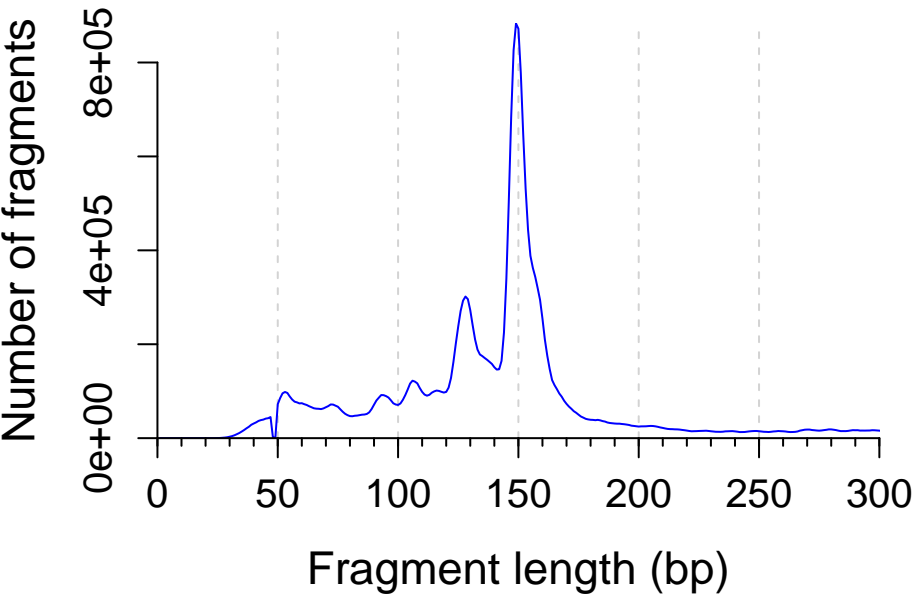

Supplement: Supplementary file 2 — Supplementary Information 2. [file 41598_2021_82842_MOESM2_ESM.zip › Eriksson_Clark_Supplemental_Code/Output_Examples/Length_histogram.111P_1_WT_50U.pdf]

# 111P\_1\_WT\_50U

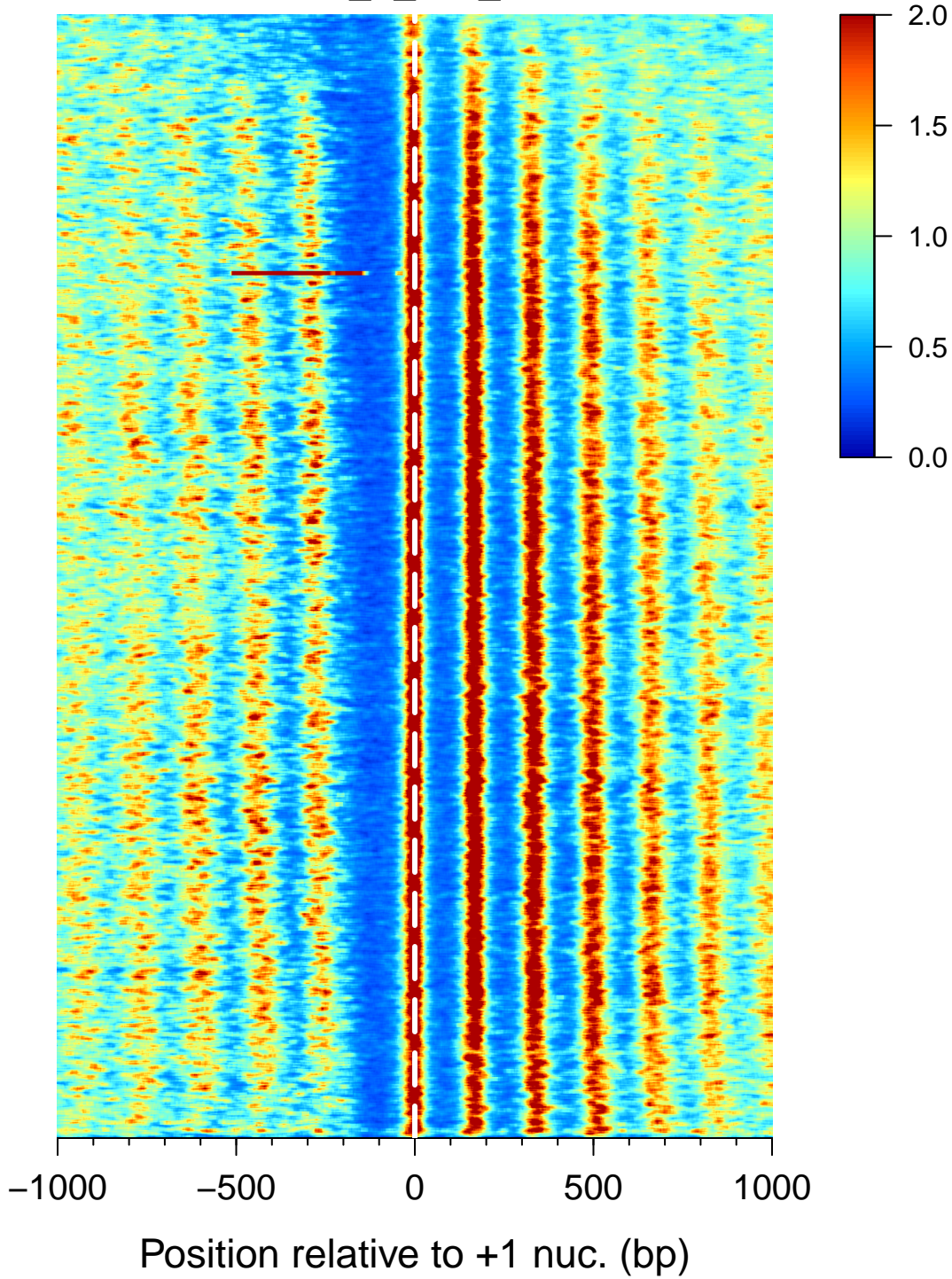

Supplement: Supplementary file 2 — Supplementary Information 2. [file 41598_2021_82842_MOESM2_ESM.zip › Eriksson_Clark_Supplemental_Code/Output_Examples/Heatmap_Dyads_Plus1.111P_1_WT_50U.120_180.Rpb3_sort.pdf]

111P\_1\_WT\_50U

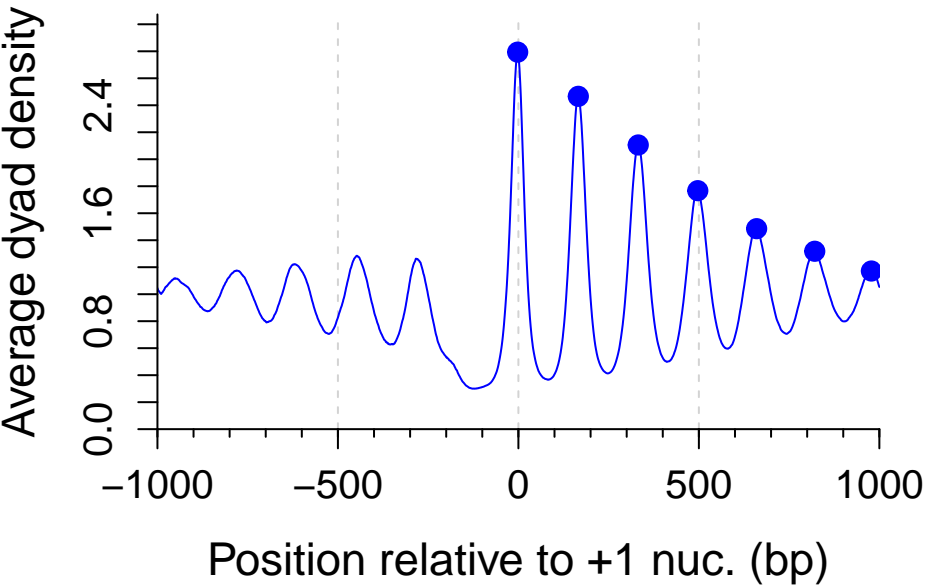

111P\_1\_WT\_50U

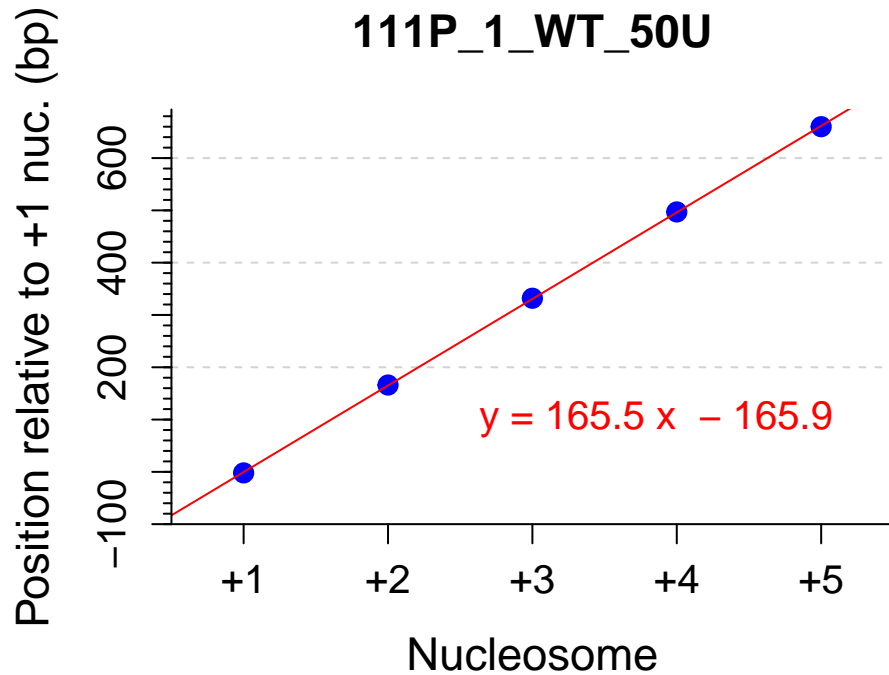

Supplement: Supplementary file 2 — Supplementary Information 2. [file 41598_2021_82842_MOESM2_ESM.zip › Eriksson_Clark_Supplemental_Code/Output_Examples/Regression_Plus1.111P_1_WT_50U.120_180.pdf]
